# 18REST: a short RIASEC-interest measure for large-scale educational and vocational assessment

**DOI:** 10.1186/s41155-018-0086-z

**Published:** 2018-02-07

**Authors:** Rodolfo Augusto Matteo Ambiel, Nelson Hauck-Filho, Leonardo de Oliveira Barros, Gustavo Henrique Martins, Loes Abrahams, Filip De Fruyt

**Affiliations:** 1 Universidade São Francisco, Waldemar César da Silveira Street, 105, Jardim Cura D’ars, Campinas, SP 13045-510 Brazil; 2 Ghent University, Ghent, Belgium; 3 Institute Ayrton Senna Chair, Ghent University, Ghent, Belgium; 4 Instituto Ayrton Senna, São Paulo, Brazil; 5 Universidade São Francisco, Waldemar César da Silveira Street, 105, Jardim Cura D’Ars, Campinas, SP 13045-510 Brazil

**Keywords:** RIASEC, Large-scale assessment, Short assessment, Sex differences, Twenty-first century skills

## Abstract

The construction of the 18REST, a short 18-item inventory to describe students’ position on John Holland’s RIASEC interest types, is documented. The instrument is meant to be used in large-scale assessment in education and on the labor market, supplementing information on school achievement and social-emotional skills. This research was carried out in Brazil, initially with two independent samples composed by adolescents and adults. The 18REST’s psychometric properties are compared to those of the more extended RIASEC item pool and confirmed in a new independent undergraduate sample. Despite differences between genders were found as expected, invariance measurement across gender was indicated. Different ways to use the 18REST in large-scale assessment are discussed.

## Backgound

The past 5 years witnessed a reviving attention for the construct of “interests” as a key individual differences variable for the understanding of educational and vocational outcomes (Nye, Su, Rounds, and Drasgow, 2012; Rounds and Su, 2014). Interests made their comeback in the Big Four of individual differences, next to psychometric intelligence, personality traits, and values. Individual differences in psychometric intelligence and personality traits are usually conceptualized as basic tendencies and considered as building blocks of more malleable constructs such as competencies or skills (De Fruyt, Wille, and John, 2015; Hoekstra and Van Sluijs, 2003). Intelligence and traits mainly describe *how* people will act or perform. The domains of values and interests, on the other hand, are more conceptualized as characteristic manifestations, and better describe *what* individuals will do, indicating domains (e.g., study majors) or areas (e.g. vocational sectors) in which people give expression to their abilities and traits. Together, these four sets of distinct constructs form powerful tools to understand how students learn and develop, and later navigate as job applicants on the labor market or develop their careers as incumbents in organizations.

Education policy-makers consistently plead for almost two decades now that education should also explicitly develop students’ social-emotional skills (also called twenty-first century skills), in addition to more traditional educational achievement indicators such as knowledge of math, languages, and sciences (Lipnevich, Preckel, and Roberts, 2016). Indeed, twenty-first century skill advocates (Trilling and Fadel, 2009) argue that students need to possess a variety of skills such as collaboration, appreciation of diversity, leadership, and innovative behavior in order to deal with challenges of the twenty-first century. Students are preparing for jobs one cannot even think of today, and they will have to work longer, underscoring the need of life-long learning and development (Noe, Clarke, and Klein, 2014). John and De Fruyt (2015) argued and demonstrated that personality descriptive models, such as the five-factor model (FFM) of personality, can be used to help structuring this broad field of social-emotional skills, with some skills also requiring building blocks of models of psychometric intelligence. For example, a twenty-first century skill like ‘critical thinking’ involves facets of the trait of openness to experience and aspects of cognitive functioning. Traits and cognitive constructs hence form core building blocks of social-emotional skills.

The link between interests and social-emotional skills is that interest patterns play a key role in individuals’ educational or vocational decisions, which in its turn might stimulate the development of specific social-emotional skills associated with that field. It is worth noting that interests are also an expression of environments where people can perform their preferred activities. Thus, in the same environment chosen to perform one’s interest, other people with similar patterns of social-emotional skills probably will provide role models for stimulate their development. An example of how profound interest differences between genders might lead to different educational decisions is the larger percentage of males enrolled in the so-called STEM (Science, Technology, Engineering and Math) majors. The meta-analysis of Su, Rounds, and Armstrong (2009) shows interest *d*-effect size differences of .36 for science, .34 for mathematics, and 1.11 for engineering between men and women, which are likely to lead to gender differences in social-emotional skill levels particularly associated with these interests. Alternatively, interests might also be directly relevant. Taxonomies of twenty-first century skills explicitly mention various content domains, such as ICT (informatics, computers and technology) literacy, green and sustainable behaviors, financial literacy, and entrepreneurship, just to name a few, as domains in which particular skills have to be learned and developed. These particular fields are probably better represented by models describing vocational interests than by personality-based social-emotional skill taxonomies. Furthermore, learning and working in a content domain that ‘fits’ with what one prefers is assumed to be motivating, and there is abundant evidence that Person-Environment fit for interests is associated with increased learning and job satisfaction (Holland, 1997). In their meta-analyses, Nye et al. (2012) showed a baseline estimate between interest congruence and academic performance of .32 and a correlation of .36 for work performance. Interests are thus also directly relevant for the learning and educational context by substantially explaining educational achievement. Therefore, in addition to describing or measuring students’ social-emotional skill levels, policy-makers also need to pay attention to interest differences among students, in order to make an adequate evaluation of the employability of a student population and its connectivity to the labor market (Brunello and Rocco, 2017; Wang and Wanberg, 2017).

Taken together, it is clear that, for several evaluation purposes, an assessment of students’ standing on twenty-first century skills will encompass a description of their acquired skill levels in association with a portrayal of their interest patterns, providing insight in the domains and areas in which they want to express and use their skills. In the past years, considerable efforts have been made already to develop social-emotional skill measures (Lipnevich et al. 2016; Primi, Santos, John, and De Fruyt, 2016) that are applicable in large-scale assessments in education. Primary requirements for such instruments are that they not only demonstrate construct and predictive validity, but also that they are reliable, easy to administer, short, and that they show measurement invariance across subgroups of the population, allowing for meaningful comparisons across groups. Zanon et al. (The role of socioeconomic status, language proficiency and grade-age correspondence in recovering personality strucutre in large-scale educational assessment in adolescence, in preparation) examined for example whether a personality-based social-emotional skill measure such as SENNA 1 (Primi et al. 2016) demonstrated measurement equivalence across groups differing in social-economic status and language proficiency. Most available interest assessment tools are designed to be used for self-exploratory purposes, helping students discovering and expanding their interest patterns, and therefore most RIASEC measures use a large set of interest items, often referring to activities that people enjoy, occupations they want to try, or skills, competencies, and characteristics they might have. However, for large-scale educational evaluation purposes, these extensive self-exploration inventories are inappropriate. For these applications, a short interest scale is warranted, one that is psychometrically sound and that works well for different subgroups in the population. The present paper will describe the process of constructing such a short measure, entitled the 18REST (a pun intended to sound next to “interest”, using the final number of items of the instrument), representing each of the six RIASEC interest types by three items.

## Representation of interests by RIASEC

Holland’s RIASEC model (1997) has been the dominant paradigm the past 50 years to describe people’s interests. Holland argues that individuals’ interest patterns can be best described in terms of their resemblance to six major interest types, i.e., realistic, investigative, artistic, social, enterprising, and conventional. The *realistic* interest type refers to preferences for technical or outdoor activities and occupations, involving the use of equipment and technology and requiring manual and hands-on skills. The *investigative* type groups interests in thinking and research activities and vocations, dealing with theory-building, abstract problem-solving, and science-methodology skills. The *artistic* interest type refers to preferences for creating and developing new things, where beauty and design are key ingredients. The *social* interest type refers to preferences regarding interacting with people, such as educating, training, caring, and nursing activities. The *enterprising* type represents likability for action and doing, expressed in activities such as implementing, organizing, and leading. Finally, the *conventional* interest type groups preferences about the correct application of rules and standards, articulated in vocations like accountant or quality controller. These six types have varying degrees of dependency and are best represented in a circular hexagonal structure, also called the RIASEC calculus, with some types adjacent to each other (e.g., R and I, I and A), others taking alternate positions, with another type in-between letters (R and A, I and S), while there are also types taking opposite positions relative to each other (R versus S, describing “things/objects” versus “people”, I versus E, opposing “thinking” versus “doing”, and finally A versus C, describing “creation” versus “rule-application”). There is support for the circular order of the types (Tracey and Rounds, 1993) and for counseling purposes an individual’s interest pattern is usually described using a three-letter code, reflecting the individual’s primary, secondary, and tertiary interest fields. Rules are defined on how to deal with ties (equal scores on multiple interest types) for composing the letter codes, although the number of ties is often small given the large number of items per interest type.

**Fig. 1:**
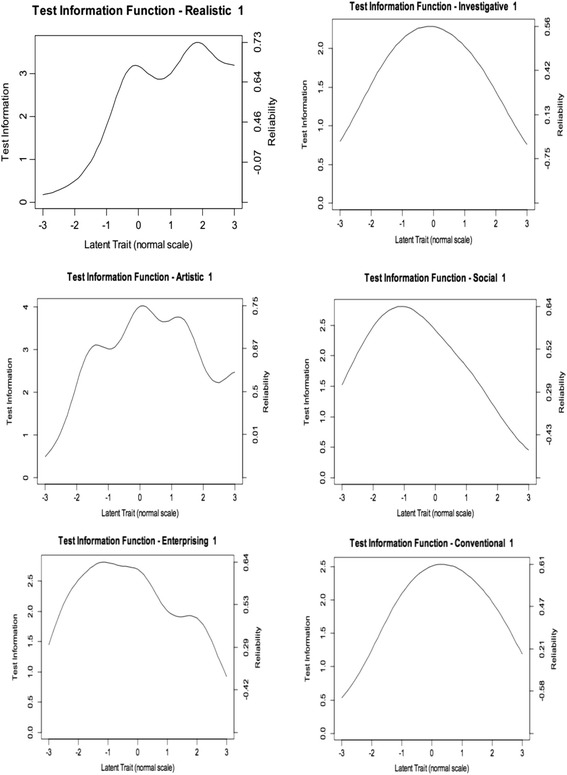
Test and item information functions

An eloquent feature of the model is that RIASEC types are also useful to denote characteristics of environments (Holland, 1997). Consequently, the model has been successfully used to describe educational majors and vocations. Its ability to explore and examine “fit” between a student’s interest pattern and features of educational majors or vocations substantially contributed to its impact in the fields of student and career counseling. The commensurate assessment of interests at the level of the individual *and* the environment enabled to examine whether RIASEC fit or congruency predicted educational or vocational outcomes better than relying on information from the person or situation separately (Kristof-Brown, Zimmerman, and Johnson, 2005). To facilitate such comparisons, all US labor force occupational titles were described in terms of their RIASEC resemblance (O*NET Resource Center, 2012). Wille and De Fruyt (2014) empirically demonstrated that these US-based O*NET descriptions align well with RIASEC descriptions provided by incumbents in a different country (Belgium) relying on the 84-item Position Classification Inventory (PCI; Gottfredson and Holland, 1984). There is thus first evidence that these O*NET descriptions also accurately reflect job characteristics in other economies and cultures. The availability of such rich and extensive descriptions in the O*NET database brings substantial information to the educational and labor market policy debate, beyond data provided by the assessment of social-emotional skills, educational attainment and interest profiles of students.

Another relevant issue is that relationships between Holland types and the big five model are well known. In a meta-analyses of 24 studies, Larson, Rottinghaus, and Borgen (2002) found substantial relations both for men and women, artistic-openness, enterprising-extraversion, social-extraversion, investigative-openness, and social-agreeableness, with coefficients ranging from .19 to .48. In another study, Wille and De Fruyt (2014) examined a sample of 266 college alumni twice, in an interval of 15 years. Among other results, they noticed that increases in extraversion over the years were associated with increases in social and enterprising characteristics, increases in conscientiousness were associated with increases with enterprising interests, and decreases in openness were related to decreases in artistics interests.

## Assessing RIASEC

Several instruments have been developed to assess resemblance with the RIASEC interest types, almost all variants of John Holland’s self-directed search measure (SDS; Holland, 1979). The SDS has been primarily developed as a tool for the self-exploration of interests. SDS measures typically list a broad series of activities and vocational titles that individuals may like or want to try out, supplemented with skills and characteristics that further define the RIASEC interest domains. The SDS family of tools has been successfully distributed and implemented world-wide, including Brazil. For example, the Self-Directed Search Career Explorer (SDS: CE; Holland, Fritzsche, and Powell, 1994) has been translated and adapted by Primi, Mansão, Muniz, and Nunes (2010) for adolescents. Teixeira, Castro, and Cavalheiro (2008) developed a 48-item version for adolescents and adults, whereas Mansão and Noronha (2011), and Meireles and Primi (2015) developed and validated a 154-item RIASEC measure for adolescents. Given their exploratory nature, SDS-based measures refer to a broad set of characteristics of the individual or activities and occupations that he or she may want to try out. Instruments are hence long and not directly useful for large-scale assessment. In addition, the content covered by interest tools is relatively time-bound: activities and vocation titles quickly change, and what is an appropriate item today may sound outdated 10 years later.

The first objective of the present paper is to assemble a new and timely RIASEC item pool covering activities that are relatively independent of an individual’s abilities or diploma. A second aim is to develop from this set a short RIASEC inventory useful for large-scale educational and labor market assessment. This short version’s psychometric characteristics, scale parameters, order of types, and measurement invariance will be examined.

## Methods

### Item compilation strategy

An initial set of interest items was generated by a group of 77 undergraduate students as part of a course in psychometrics organized at a university in the state of São Paulo, Brazil. All of the items were written in Portuguese. Students first studied a selected set of papers to get familiar with Holland’s model and the content of the RIASEC types. They were then split up in 12 supervised groups and instructed to generate 20 to 30 items for each Holland type, resulting in a first set of 341 items. In a second step, items were evaluated by three psychologists including the lecturer-in-charge, all three experts in Holland’s theory and RIASEC. They independently judged each item’s (1) grammar and wording, (2) quality to function as a psychological assessment item, and (3) representativeness of the specified RIASEC type. Scores varied from 1 to 3, with higher scores indicating a better item. Only items receiving a score of 3 on all criteria were retained, resulting in a set of 54 items, distributed across types as follows: realistic: 10 items, investigative: 8 items, artistic: 9 items, social: 8 items, enterprising: 10 items, and conventional: 9 items.

### Participants

A sample of adolescents (sample 1: *N* = 241) and an adult sample (sample 2: *N* = 473) were administered the 54-item set. The adolescent sample had a mean age of 16.32 years (SD = 1.05; with 50.6% males [*N* = 119]). Education was as follows: 36.7% were first graders, 32.1% were second graders, and 31.3% were third graders, all attending high school. The adult sample had a mean age of 29.48 years (SD = 10.21; 67% females [*N* = 317]). Regarding education level, 0.8% had only completed elementary school, 14.8% completed high school, 63.6% were attending college, and 20.8% had completed college. The two samples were merged for the instrument-developing analyses.

A third sample was available for cross-validation and measurement invariance analysis. The short form was administered to 292 undergraduate students, ranging in age from 18 to 64 years (*M* = 23.37, SD = 8.44), including 59.2% females (*N* = 173). Sample composition was intentionally heterogeneous, including undergraduates from more than 50 different majors, to represent participants with largely varying interests.

### Procedures

This work is a part of a broader project that was submitted and approved by an Ethical Committee. Sample 1 was collected in a pen-and-paper style, in one high school in the southwest of Brazil. Parents of all participants signed an informed consent term before the adolescents filled the instrument. Samples 2 and 3 were collected on line, in a Google Forms platform, and adults and students only accessed the protocol after agreeing with an informed consent term.

### Data analytic strategy

The possibility of fitting a factor model to the aggregated data of the first two samples was evaluated using the Kaiser−Meyer−Olkin (KMO) index. Decision-making as to factor retention was guided by parallel analysis with data permutation (Timmerman and Lorenzo-Seva, 2011) and the Hull method (Lorenzo-Seva, Timmerman, and Kiers, 2011). Given the ordered categorical nature of variables, exploratory factor model parameters were estimated using robust weighted least squares mean − and variance−adjusted (WLSMV), as this has been recommended in recent simulation studies for the analysis of Likert−type data (Asún, Rdz-Navarro, and Alvarado, 2015). Information functions of each resulting scale were inspected to examine the achieved latent variable coverage. Analyses were performed using Mplus 7.11 (Muthén and Muthén, 2014) and the psych package (Revelle, 2014) for R. Additional analyses included linear correlations and *t* tests for independent samples, conducted using SPSS 21, and tests for significance of the difference between correlation coefficients, performed using the online calculator available on http://vassarstats.net/rdiff.html. Psychometric properties were re-evaluated in a third sample using a constrained confirmatory factor model followed by an analysis of measurement invariance across gender in a combination of all three samples. These last analyses were planned in an attempt to (a) investigate the stability of the 6-factor structure of the 18REST in an additional dataset independent from the samples used in the scale development phase, and (b) inspect the stability of the estimated item parameters according to gender. Measurement invariance is a fundamental property for an instrument designed to provide group comparisons, as it ensures that mean differences in scores reflect true differences in the latent variable instead of systematic biases in the items.

## Results

### Factoring the 54-item set

The KMO index was .91, underscoring the factorability of the data. Both methods of factor retention—Hull method and parallel analysis with data permutations—suggested the existence of six factors underlying the data, so we decided to proceed estimating parameters for a 6-factor model. The initial analysis included the complete 54 − item pool and results are presented in Table 1. Despite the significance of the chi-square test, the 6-factor model achieved a reasonable fit to the data, χ^2^(1122) = 8530.06, *p* < .001, RMSEA = .096, CFI = .962, TLI = .952. Nearly all items (but item 43) revealed to be unidimensional indicators of the six factors, approximating simple structure with only small cross-loadings on other factors. Internal consistency estimates (alpha coefficients) for the scales were excellent, ranging from .86 to .92.

**Table 1:** WLSMV exploratory factor analysis of 54 initial item pool

	R	I	A	S	E	C
Int 1	*.95*	−.06	−.01	.06	−.03	−.02
Int 2	*.90*	−.04	.03	.02	.01	−.04
Int 3	*.92*	−.00	.05	.03	−.04	−.01
Int 4	*.91*	.05	−.03	−.03	.03	.01
Int 5	*.84*	.10	−.04	−.08	.05	−.05
Int 6	*.80*	−.03	.01	−.02	.03	.07
Int 7	*.69*	−.04	.06	.14	−.06	.01
Int 8	*.62*	−.02	.04	.01	.08	.05
Int 9	*.52*	.00	−.06	−.07	.40	.06
Int 10	*.58*	.11	.03	−.11	.09	.03
Int 11	.02	−.04	−.07	−.10	*.89*	.02
Int 12	.03	−.06	−.05	−.11	*.87*	.02
Int 13	.05	.13	.06	.05	*.91*	−.15
Int 14	.08	.13	.03	.06	*.93*	−.13
Int 15	.03	.07	.04	.07	*.67*	−.01
Int 16	.13	.03	.02	.02	*.78*	−.08
Int 17	.17	−.19	−.11	.05	*.55*	.15
Int 18	.07	−.33	.11	−.01	*.62*	.11
Int 19	.23	−.16	−.05	−.08	*.48*	.22
Int 20	.10	−.31	.19	.08	*.49*	.06
Int 21	.07	−.01	−.06	*.89*	.03	−.07
Int 22	.05	.10	−.11	*.91*	−.02	−.02
Int 23	.00	−.06	.09	*.80*	.01	−.05
Int 24	.06	.08	−.10	*.84*	.05	.04
Int 25	.04	−.14	.13	*.77*	−.00	.02
Int 26	.08	.04	.08	*.66*	.01	.19
Int 27	.07	.02	.06	*.72*	.03	.07
Int 28	.14	.04	.07	*.58*	−.06	.10
Int 29	.03	−.10	*.82*	.07	−.05	.05
Int 30	.06	.02	*.82*	.03	.07	−.01
Int 31	.08	.00	*.88*	−.07	.05	.03
Int 32	.01	.05	*.73*	−.01	.02	.05
Int 33	.08	−.02	*.67*	−.17	.14	−.03
Int 34	.20	.19	*.70*	.02	−.06	−.08
Int 35	.12	−.09	*.79*	.06	.03	.03
Int 36	.19	.17	*.64*	−.16	−.04	.10
Int 37	.17	.22	*.66*	.07	−.02	−.13
Int 38	.03	*.85*	−.02	−.04	−.01	.21
Int 39	.16	*.86*	−.01	−.02	.06	.04
Int 40	.22	*.82*	−.00	.06	−.01	.03
Int 41	.09	*.53*	.11	.06	−.06	.36
Int 42	.39	*.56*	−.01	.09	.03	−.12
Int 43	**.** *47*	*.50*	.07	−.02	−.00	.02
Int 44	.00	*.49*	.13	.15	.11	.19
Int 45	.06	*.48*	.02	.19	.01	.34
Int 46	.03	.09	.00	.10	−.02	*.80*
Int 47	.03	−.05	.02	−.01	.02	*.86*
Int 48	.12	−.04	−.00	−.02	.07	*.79*
Int 49	.08	−.10	−.01	.05	−.01	*.76*
Int 50	.08	.07	−.01	.30	−.01	*.68*
Int 51	.01	.03	.03	−.08	.21	*.66*
Int 52	.13	.08	−.05	−.11	.25	*.63*
Int 53	.07	.26	.05	.21	−.05	*.57*
Int 54	.15	−.10	.08	.10	.08	*.47*
Alpha coefficient	.92	.86	.89	.89	.89	.89

Note. Factor loadings ≥ .40 are in italics. *R* realistic, *I*  investigative, *A* artistic, *S* social, *E* enterprising, *C* conventional

### Short measure and psychometrics

As our main interest was the development of a brief RIASEC inventory, the next step was the selection of items for this short instrument. Three criteria were simultaneously used to guide the selection process: (a) maximizing content coverage of each vocational interest by avoiding redundant items; (b) selecting items with large factor loadings, maximizing discriminant validity and reliability; and (c) retaining a similar small number of items per dimension, to obtain a balanced assessment of each type. Finally, a set of 18 items was retained, three per RIASEC dimension. A 6-factor exploratory structural equation analysis of this 18-item set resulted in an excellent fit to the data, χ^2^(60) = 172.29, *p* < .001, RMSEA = .051, CFI = .993, TLI = .981. Estimated parameters for this factor model are presented in Table 2. Internal consistency estimates (alpha coefficients) were adequate considering that each scale had only three items, with values ranging from .68 to .81. The six factors were clearly interpretable as Holland’s vocational interest types. The abbreviated instrument showed high positive correlations of *r*_R_ = .94, *r*_I_ = .87, *r*_A_ = .93, *r*_S_ = .90, *r*_E_ = .94, and *r*_C_ = .88 with its 54-item parent.

**Table 2:** Final 18-item, 6-factor solution

Item code	Item label	R	I	A	S	E	C
INT1	Operate machines for producing machine parts.	*.97*	−.00	−.01	.04	−.03	−.03
INT4	Perform maintenance on machines and tools.	*.80*	.06	−.01	−.03	.03	.04
INT10	Calculate the area of geometric figures.	*.46*	.24	−.02	−.07	.12	.06
INT38	Read scientific papers and books.	−.21	*.62*	.00	.09	−.05	.19
INT42	Perform analyses and lab experiments.	.08	*.74*	.00	.04	.03	−.14
INT43	Explain natural physical phenomena.	.16	*.70*	.08	−.08	−.00	.00
INT30	Participate in the designing of scenarios for theater pieces.	−.03	.06	*.74*	.09	.02	.01
INT31	Perform an artistic presentation to an audience.	−.03	.00	*.98*	−.06	.01	−.02
INT32	Sing in a choir.	.07	−.01	*.64*	.13	−.03	.03
INT26	Provide social services in communities and neighborhoods.	−.03	.04	.02	*.73*	.00	.14
INT27	Provide guidance to individuals, groups or population about health and well-being.	.02	−.03	−.01	*.82*	.03	.01
INT28	Be available to help people.	−.08	.04	.04	*.64*	−.01	−.05
INT11	Take part in strategic planning for companies.	−.02	.02	−.09	.03	*.93*	−.01
INT13	Manage goals and performance of work teams.	.03	.01	.04	.13	*.70*	−.03
INT18	Negotiate with customers.	.04	−.13	.10	−.07	*.58*	.15
INT48	Supervise the compliance with laws.	.08	−.00	.01	.06	−.00	*.76*
INT52	Analyze national and international economic scenarios.	−.04	.14	−.04	−.07	.20	*.71*
INT54	Archive important documents and files.	.17	−.08	.06	.16	−.00	*.53*
Alpha coefficient		.78	.68	.81	.74	.74	.71

*Note.* Factor loadings ≥ .40 are in italics. *R* realistic, I investigative, *A* artistic, *S* social, *E* Enterprising, C = Conventional

To perform a more in-depth inspection of reliability, we then conducted test information curve analyses, as these reveal the amount of information that is provided by the scale along the latent continuum. Results are depicted in Fig. 1. Plots represent the information (i.e., precision) of each scale across distinct levels of the latent variable considering the population of individuals. Peaks indicate the location where the scales achieve their maximum reliability or, in other words, where they provide an ordering of individuals with the smallest amount of error. Test information curves further indicate the quality of content coverage, which can be seen from the spread of the curve along the latent trait continuum. Once again, brevity did not hamper precision or content coverage, as test and item functions were distributed along a reasonable area of the latent variable representing each vocational interest. Despite peaks reaching no higher than .75, precision was still impressive given that scales contain only three items.

**Fig. 2:**
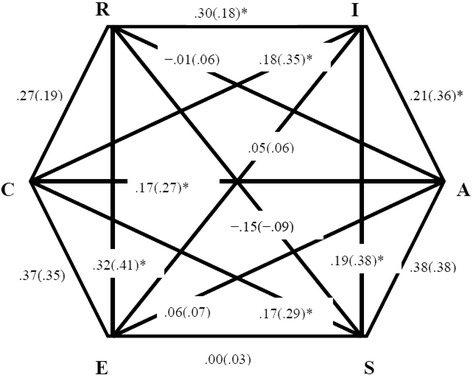
The observed circular order for the 18-item and the 54-item (inside parentheses) instruments

It was further evaluated whether the empirical structure of between-type correlations of the short form matched the presumed RIASEC order. Holland’s model predicts that adjacent dimensions will correlate stronger than alternate types, and the latter should show stronger correlations than opposite RIASEC types. We examined these assumptions separately for gender and for the samples of adolescents and adults. Results can be found in Fig. 2. For the sake of comparison, we included the correlation coefficients for the brief and the extended (inside parentheses) inventories. We also tested for the statistical significance of the difference between correlations as a means for identifying moderation effects (significant differences are marked with “*”). The pattern of correlations between dimensions was only moderately consistent with the expectations, especially in the short version (18REST) of the inventory. An exception that is worth mentioning was E, which tended to be more correlated to R (*r*_18-item_ = .32, *r*_54-item_ = .41) than to S (*r*_18-item_ = .00, *r*_54-item_ = .03).

### Gender and age differences

Gender and age (adolescent versus adult sample) score differences on the 18REST are displayed in Tables 3 and 4. The aggregated sample showed moderate-to-large gender differences for five RIASEC scales, except conventional. Men scored higher on realistic (large effect, as defined by Cohen, 1988), investigative (small effect), and enterprising (large effect) interests, whereas women obtained higher scores on artistic (large effect) and social (large effect) interests. These patterns were observed in both adolescents and adults, with only two exceptions: adult males had higher scores on both the investigative and conventional scales, but this was not the case in adolescents. Considering age (adolescents versus adults), adults obtained higher scores (small to large effect sizes) for all RIASEC types, except enterprising.

**Table 3:** Between-sex mean comparisons (standard deviations within parentheses) for the disaggregated and the aggregated sample

	Adolescents			Adults			Total sample		
	Men	Women	d	Men	Women	d	Men	Women	d
R	2.19(.99)	1.45(.64)	.91*	3.03 (1.12)	1.85 (.77)	1.25*	2.66 (1.14)	1.74 (.75)	.97*
I	2.83 (1.09)	2.89 (1.20)	.05	3.45 (.87)	3.02 (.89)	.49*	3.17 (1.02)	2.99 (.98)	.18*
A	1.73 (.97)	2.71 (1.30)	.86*	2.54 (1.03)	3.02 (1.06)	.46*	2.18 (1.08)	2.93 (1.14)	.68*
S	2.95 (1.06)	3.71 (1.01)	.73*	3.47 (.88)	3.97 (.77)	.60*	3.24 (1.00)	3.90(.85)	.71*
E	3.36 (1.05)	2.91 (1.14)	.41*	3.54 (.88)	3.08 (.99)	.49*	3.46 (.96)	3.03 (1.03)	.43*
C	2.36 (1.06)	2.48 (1.13)	.11	2.96 (.90)	2.61 (.95)	.38*	2.70 (1.02)	2.57 (1.01)	.13

*Note*. *p < .05

**Table 4:** Between-sample mean comparisons (standard deviations within parentheses)

	Adolescents	Adults	d
R	1.83(.91)	2.23 (1.06)	.40*
I	2.86 (1.14)	3.16 (.90)	.29*
A	2.21 (1.25)	2.86 (1.08)	.56*
S	3.32 (1.10)	3.81 (.84)	.51*
E	3.14 (1.11)	3.23 (.98)	.09
C	2.42 (1.10)	2.73(.95)	.30*

Note. *p < .05

### Cross-validation of structural properties

Key psychometrics of the 18REST were re-examined in a new sample of undergraduates (sample 3). This time, a constrained confirmatory factor model was specified by allowing each item to load only on its expected factor. Despite being prohibitive, this model with no cross-loadings yielded a good approximate fit to the data, χ^2^(60) = 282.18, *p* < .001, RMSEA = .068, CFI = .932, TLI = .913. As can be observed in Table 5, factor loadings and internal consistency estimates were slightly smaller than in the combined samples used for scale development. Nonetheless, factor loadings were still remarkably large in magnitude, and internal consistency estimates, with only two exceptions, fell above the .70 cut-off.

**Table 5:** Confirmatory factor analysis of data from 292 undergraduate students

Item code	Item label	R	I	A	S	E	C
INT1	Operate machines for producing machine parts.	*.87*					
INT4	Perform maintenance on machines and tools.	*.91*					
INT10	Calculate the area of geometric figures.	*.72*					
INT38	Read scientific papers and books.		*.44*				
INT42	Perform analyses and lab experiments.		*.67*				
INT43	Explain natural physical phenomena.		*.82*				
INT30	Participate in the designing of scenarios for theater pieces.			.76			
INT31	Perform an artistic presentation to an audience.			.85			
INT32	Sing in a choir.			.59			
INT26	Provide social services in communities and neighborhoods.				*.81*		
INT27	Provide guidance to individuals, groups or population about health and well-being.				.72		
INT28	Be available to help people.				.72		
INT11	Take part in strategic planning for companies.					*.84*	
INT13	Manage goals and performance of work teams.					.57	
INT18	Negotiate with customers.					.72	
INT48	Supervise the compliance with laws.						.57
INT52	Analyze national and international economic scenarios.						.72
INT54	Archive important documents and files.						.58
Alpha coefficient		.78	.62	.72	.71	.70	.59

Note. Factor loadings ≥ .40 are in italics. *R* realistic, *I* investigative, *A* artistic, *S* social, *E* enterprising, *C* conventional

### Measurement invariance analysis

It was also investigated whether the item parameters of the 18REST were invariant across gender. The initial step was inspecting whether the constrained 6-factor model fitted the aggregate data in the three samples combined. Model fit was reasonable, but not optimal, χ^2^(120) = 975.78, *p* < .001, RMSEA = .084, CFI = .909, TLI = .884. An inspection of modification indices helped identifying the cause of misfit: a residual correlation between items 1 and 2 of the R dimension, which have similar content. A model that included such residual correlation had an excellent fit to the data, χ^2^(119) = 489.63, *p* < .001, RMSEA = .056, CFI = .961, TLI = .949, and was deemed adequate for the invariance analysis. Testing measurement invariance was then proceeded by allowing factor loadings, thresholds, and item residuals to differ across genders. For the sake of model identification, factor means and variances were set to 0 and 1, respectively. Once again, model fit was excellent, χ^2^(119) = 593.34, *p* < .001, RMSEA = .055, CFI = .961, TLI = .950, largely supporting measurement invariance across gender.

## Discussion

This work aimed to develop a short RIASEC measure, appropriate for large-scale assessment in education and employability evaluation. To be broadly applicable, the 18REST was constructed from a larger set of 54 items, representing activities that persons might enjoy, so that the measure can be used irrespective of previous background training or experience. Considering that scales have only three items per interest type, 18REST’s psychometric properties are good and the instrument has adequate reliabilities (ranging from .68 to .81). The information curve analyses show that items capture variance across their respective latent RIASEC dimension. Types assessed using the short version showed substantive correlations with their parent RIASEC types (all above .87), and the trimmed and 54-item set showed a comparable ordering of types, with a tendency of adjacent types on average showing the strongest correlations. The psychometric characteristics observed in the combined development samples (1 and 2) were replicated in an independent sample of undergraduates. The scales also proved measurement invariant across gender (grouping subjects from the three samples), which is a prerequisite for making meaningful gender comparisons.

Parallel to other RIASEC instruments (Su et al. 2009), large gender differences were demonstrated with men scoring higher on realistic and enterprising interests, and women scoring higher on the artistic and social types. These effects were apparent in both adolescents and adults, but in adulthood, men also scored substantially higher on investigative and conventional interests. The adult sample scored higher on five of the types, except enterprising. Effect sizes for all differences were medium to large. The large gender effects for realistic (men higher) and social (women higher) are in line with international research (Su et al. 2009). The magnitude observed for the other types is larger than those found with more extended RIASEC inventories: additional items probably help to flat gender differences to some extent, although it is difficult, if not impossible, to avoid the previously described gender patterns (Fonteyne, Wille, Duyck, and De Fruyt, 2016).

The present contribution has important utility, both conceptually and pragmatically. First, the availability of a short RIASEC measure opens new perspectives to expand twenty-first century skill models, such as those proposed by the OECD (John and De Fruyt, 2015) and Primi et al. (2016), with a model specifying the educational or vocational areas in which social-emotional skills are preferably demonstrated, practiced, and further developed. Supplemental RIASEC descriptions in large-scale educational and labor market assessments allows to connect observed social-emotional skill levels with required skill levels as described in O*NET, comparing educational output with labor market requirements. 18REST will hence enable a better monitoring of educational outcomes and evaluate student cohorts’ employability beyond achievement results (e.g., scores on math and languages) and social-emotional skill levels. These three pieces of educational deliverables are crucial to evaluate how education meets the requirements of society and the labor market at a certain moment in time. Secondly, 18REST could further be used for impact evaluation of policies to promote STEM educational majors and to attract more students to these programs. The number of students in such majors is the best direct evidence to evaluate the success of such intervention, though many students enroll in a non-STEM major, while also having interest patterns that align with STEM programs. 18REST can help detecting such latencies in large-scale assessment and advise policy-makers on how to develop this potential and especially where to find this potential. A third application is for policy-makers who may be interested in developing specific groups of skills, such as entrepreneurship skills, among students. 18REST can identify those individuals already showing primary entrepreneurial interests. Scores on the remaining five types will be additionally informative about the domains in which students would like to be entrepreneurial. For example, those with enterprising, but also artistic interests, may want to study or work in the field of (cultural) event management, organizing exhibitions or promoting dance and music festivals. Finally, an evaluation of students’ interest patterns also provides a unique opportunity to tap into students’ extracurricular activities and assemble some primary information on what students do or have been doing and developing beyond formal education curricula. Outside school learning also contributes to the capital that students bring to school and to the labor market, and is not necessarily reflected by a student’s major or his or her social-emotional skill level.

There are also limitations to the current work. First, there are multiple ways to construct a trimmed version of a larger scale. The approach applied here relied mainly on psychometric criteria. Alternatively, one could have given more weight to the representation of content captured by a RIASEC type or retain more gender-neutral items. Wille, De Fruyt, Dingemanse, and Vergauwe (2015), for example, recently proposed a facet structure within the RIASEC types, so that each facet in their model can be represented by one item in an abbreviated version. Likewise, gender-differences could be dampened probably by writing and selecting more gender-neutral items (for a more extended discussion on its feasibility see Fonteyne et al. 2016). A second limitation is the nature of the construction and validation samples, exclusively examining Brazilian adolescents and adults. Examining 18REST’s validity in English-speaking samples of adolescents and adults (and also other languages) is one of the next steps to take. A third limitation pertains to the empirical circular structure of the 18REST, which was only modestly consistent with the presumed RIASEC order. The size of correlations found in this regard could reflect the small number of indicators and the fact maybe some of the resulting scales emphasize specific domain facets.

## Conclusions

Taken together, it is clear that a description of interest patterns is a welcome and necessary supplement of information beyond social-emotional skills’ levels, given that interests define contexts in which individuals like to use and manifest their skills. In addition, interests are important from a motivational point of view for understanding the learning process, considering that interests’ fit facilitates learning. Finally, interests further help in identifying the content domains (e.g., information and computer technology, STEM, entrepreneurial activities) that are considered as key-developmental areas in current twenty-first century skill learning (Lipnevich et al. 2016). RIASEC and social-emotional skill measurement hence go hand-in-hand delivering necessary input for evidence-based policy-making. The 18REST is a valuable and promising tool to help achieving this objective.
